# Physicochemical, Thermal and Textural Characterization of Olive Pomace Oil and Argan Oil Oleogels Prepared with Different Oleogelators

**DOI:** 10.3390/gels11120997

**Published:** 2025-12-11

**Authors:** Mine Kırkyol, Ahmet Akköse, Şeyma Şişik Oğraş, Zeynep Feyza Yılmaz Oral, Güzin Kaban, Mükerrem Kaya

**Affiliations:** 1 Department of Hotel, Restaurant and Catering Services, Artvin Vocational School, Artvin Çoruh University, 08010 Artvin, Türkiye; minekirkyol@artvin.edu.tr; 2 Department of Food Engineering, Faculty of Agriculture, Atatürk University, 25240 Erzurum, Türkiye; seymasisik@atauni.edu.tr (Ş.Ş.O.); zeynep.yilmaz@atauni.edu.tr (Z.F.Y.O.); gkaban@atauni.edu.tr (G.K.); mkaya@atauni.edu.tr (M.K.)

**Keywords:** oleogel, olive pomace oil, argan oil, carnauba, DSC, texture profile

## Abstract

The physicochemical, thermal and textural properties of oleogels formed from olive pomace oil and argan oil using carnauba (KW), candelilla (CW) and sunflower (AW) waxes and their combinations (KCW: 50% carnauba + 50% candelilla wax, KAW: 50% carnauba + 50% sunflower wax, CAW: 50% candelilla + 50% sunflower wax) were investigated. The highest mean L* value, peroxide value and time of crystallization formation were found in AW oleogelator. Argan oil + AW had the lowest mean L* value. Sunflower wax differed from the other waxes in terms of major fatty acids, and oleogels containing argan oil and olive pomace oil exhibited a different major fatty acid profile from each other; in particular, there were higher values of oleic acid content in the groups where olive pomace oil was used. It was determined that KW and the oleogels containing KW had higher melting and crystallization temperatures and enthalpy values compared to other waxes. The hardness, adhesiveness, gumminess, cohesiveness and springiness values of the oleogels were affected by the oils and waxes used. The oleogels using sunflower wax were different in terms of texture profile from oleogels formed with carnauba and candelilla waxes.

## 1. Introduction

Fat plays an important role in human nutrition, as it contains elements such as vitamins, bioactive compounds and aroma components, as well as being an important source of energy. In food technology, fat is often used to improve the physicochemical and textural properties of processed foods. In addition, oil is used in the production of various food products by solidifying them through processes such as hydrogenation, interesterification and fractionation. However, the consumption of fat can have some negative effects on human health due to saturated and trans fatty acids [1]. Additionally, studies have shown that saturated and trans-fat intake increases the risk of cardiovascular disease, obesity and diabetes [2,3]. For these reasons, the development of new alternatives to obtain oil in solid form, free of saturated and trans-fatty acids, is becoming increasingly important. Oleogelation is defined as the process of converting oils into a gel-like structure with fat properties using an oleogelator. The resulting oleogels retain the properties of oils and do not form trans fats [4,5]. These aspects make oleogelation a promising method for the use of oils in solid form instead of fats in the food industry. Since the oils and oleogelators used in oleogelation are the main factors determining the properties of the formed oleogels, the physicochemical, thermal and textural characterization of oleogels to be formed using specific oils and oleogelators is very important in determining the functionality of these oleogels and in selecting their areas of application. Oleogels are classified as single component (using one oleogelator) or multi-component (using two or more oleogelators), depending on the use of oleogelators. However, multi-component oleogels are reported to show better performance due to synergistic combinations between oleogelators [6].

Commonly used oleogelators in oleogelation are vegetable waxes, mono-diglycerides, alcohols or esters of fatty acids, phospholipids and phytosterols. Among these oleogelators, waxes have an important role due to their high structuring ability, improvement of textural properties and high oil binding capacity [7]. Waxes are easily available and economical [8]. Waxes are considered effective oleogelators due to their ability to form oleogels even at low concentrations, due to their high oil binding capacity [9,10].

Carnauba wax, obtained from the leaves of the carnauba palm (*Copernicia prunifera*), is an important type of vegetable wax used in oleogelation. Carnauba wax has a hard and shiny structure, as well as a high melting point (80–85 °C) [11]. With heterogeneous properties, carnauba wax contains 84% wax esters, 6.5–9.5% fatty acids, fatty alcohols and hydrocarbons, and 6.5–10% resins [8]. This wax is highly efficient for creating strong crystal networks due to its composition. Another plant-based oleogelator that can be used in oleogelation is candelilla wax. Candelilla wax, obtained from the leaves of *Euphorbia cerifera* and *Euphorbia antisyphilitica* plants native to northern Mexico and the southwestern United States, has a more heterogeneous structure consisting of 27–35% wax esters, 7–10% free fatty acids, 10–15% free fatty alcohols and 50–65% hydrocarbons [11]. In addition, sunflower wax, obtained during the refining of sunflower oil as a byproduct, can be used as an oleogelator in oleogelation. The composition of sunflower wax includes 96% wax esters, 3% free fatty acids, 0.3% free fatty alcohols and 0.2% hydrocarbons [12]. Investigating these three specific waxes enables a comprehensive understanding of how different minor components affect the thermal and textural behavior of the oleogels.

The choice of liquid oil in oleogelation is critical, not only for the nutritional profile but also for the sustainability and economic viability of the final product. There is a growing interest in olive pomace oil, which is obtained by drying the olive pomace left over from olive oil production and then subjecting it to solvent extraction [13], because it has a composition similar to that of olive oil, especially in terms of oleic acid content (55–83%), and is more economical than olive oil. Converting olive pomace oil into an oleogel is a promising strategy for valorizing this byproduct, transforming a low-cost liquid oil into a functional solid fat replacer suitable for industrial food applications. On the other hand, argan oil, extracted from the kernels of the *Argania spinosa* tree, represents a high-value oil with exceptional nutraceutical properties. Characterized by high levels of oleic (46–48%) and linoleic (31–35%) acids, it is rich in polyphenols and tocopherols [14,15,16,17,18]. However, its liquid state limits its use in solid food formulations. Structuring argan oil into an oleogel not only expands its utility in products like bakery goods, meat products or spreads, but may also help protect its bioactive compounds against oxidation by entrapping them within the gel network. Given its potential to prevent cardiovascular disease and cancer [16,18,19,20], developing argan oil-based oleogels offers a novel approach to delivering these health benefits in a solid fat format.

The economic feasibility of oleogel production is heavily dependent on the cost of the base oil and the oleogelator. Olive pomace oil has a significant economic advantage as a byproduct of the olive oil extraction industry. In contrast, argan oil is a high-cost raw material. Oleogels prepared with argan oil can serve as carriers for bioactive compounds, targeting the functional food and nutraceutical sectors where consumers are willing to pay a premium for health benefits. Regarding the oleogelators, sunflower wax provides another avenue for cost reduction. Since sunflower wax is recovered as a waste product during the winterization of sunflower oil, its utilization not only reduces raw material costs compared to imported waxes like carnauba but can also contribute to a circular economy by reducing waste disposal efforts.

In this study, the physicochemical, thermal and textural properties of oleogels formed from olive pomace and argan oil using carnauba (KW), candelilla (CW) and sunflower (AW) waxes and their combinations (KCW: 50% carnauba + 50% candelilla wax, KAW: 50% carnauba + 50% sunflower wax, CAW: 50% candelilla + 50% sunflower wax) were investigated. Some properties of waxes and their mixes were also determined.

## 2. Results and Discussion

### 2.1. Physicochemical Properties

The color properties, peroxide value, crystallization formation and oil binding capacity of oleogels and waxes are provided in Table 1. The highest average value for L* value, an indicator of brightness, was determined in sunflower wax (AW), while the lowest average values were determined in %50 carnauba + %50 candellilla waxes (KCW) and %50 carnauba + %50 sunflower waxes (KAW). On the other hand, AW oleogelator showed lower average a* and b* values compared to other oleogelators (*p* < 0.05) (Table 1). However, the highest mean value for b*, an indicator of yellowness, was determined in carnauba wax (KW), followed by candellilla wax (CW) and KCW and KAW oleogelators, respectively (Table 1).

Among the oleogels, the lowest mean L* value was determined in argan oil + AW (AA) oleogel, while the highest mean L* values were determined in olive pomace oil + CW oleogel (PC), olive pomace oil + KAW (PKA) and argan oil + CAW (ACA) oleogels (*p* < 0.05). However, the highest mean a* value was determined in AA oleogel, and the lowest a* value was determined in olive pomace oil + CAW (PCA) oleogel (Table 1).

**Figure 1:**
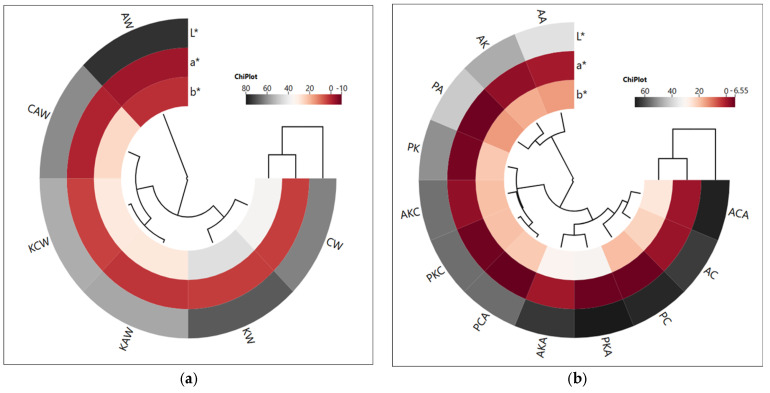
The cluster analysis of a heat map showing the relationship between waxes and color parameters (**a**) and between oleogels and color parameters (**b**) (AK: argan oil + KW oleogel, PK: olive pomace oil + KW oleogel, AC: argan oil + CW oleogel, PC: olive pomace oil + CW oleogel, AA: argan oil + AW oleogel, PA: olive pomace oil + AW oleogel, AKC: argan oil + KCW oleogel, PKC: olive pomace oil + KCW oleogel, AKA: argan oil + KAW oleogel, PKA: olive pomace oil + KAW oleogel, ACA: argan oil + CAW oleogel, PCA: olive pomace oil + CAW oleogel).

The cluster analysis of the heat map showed that two main clusters were formed depending on waxes, and the AW group was in a different cluster. This result shows that AW clearly differs from other waxes in terms of color properties (Figure 1a). The oleogel groups formed using argan and olive pomace oils were also divided into two main clusters. The first cluster included AA, AK and PA groups, and AK and PA groups showed a closer correlation. On the other hand, ACA, AC, PC, PKA and AKA groups were in the same cluster and showed relatively higher L* values. This result shows that the oleogels in this cluster are brighter and have a closer correlation with each other (Figure 1b). These results show that the oils and oleogelators used in the study have a significant effect on the color properties of the oleogels prepared. As a matter of fact, it has been reported in some other studies that different oils or oleogelators used in oleogel preparation affect the color properties [21,22].

The peroxide value serves as a primary indicator of the initial stage of lipid oxidation. In this study, the lowest average peroxide value was determined as 1.99 ± 0.01 meq O_2_/kg oil in CW and the highest value as 134.93 ± 0.47 meq O_2_/kg oil in AW, and the use of AW in binary mixtures caused an increase in the peroxide value (Table 1). The high peroxide value in AW indicates that the commercial sunflower wax used in this study had undergone significant oxidation before oleogelation, likely due to the conditions during its production (refining, winterization) or storage. On the other hand, the lowest average peroxide value was determined in AC oleogel, and the highest peroxide value was determined in PCA oleogel. In a study by Lim et al. [23], carnauba, candelilla and beeswax were used to form oleogels from canola oil, and the oleogels with candelilla wax were reported to have the lowest peroxide values.

Higher peroxide values were determined in olive pomace oil oleogels prepared using the same oleogelator than in argan oil oleogels, which is probably due to the higher peroxide value of olive pomace oil. While the peroxide value for olive pomace oil was determined as 5.47 ± 0.09 meq O_2_/kg oil within the scope of the study, this value was determined as 0.59 ± 0.01 meq O_2_/kg oil for argan oil. However, regardless of the oil type, samples containing AW consistently displayed the highest oxidation levels due to the poor initial quality of the wax (Table 1). This was not merely a dilution effect; the oxidized wax likely acted as a pro-oxidant, introducing a high concentration of free radicals and hydroperoxides into the system. These oxidation products serve as seeds that accelerate the oxidative degradation of the unsaturated fatty acids present in the olive pomace and argan oils, thereby reducing the overall oxidative stability of the AW-based oleogels.

The highest time for crystallization formation was determined in AW, followed by KAW. On the other hand, the lowest time for crystallization formation was observed in CAW, with 210 s (Table 1). In general, higher crystallization formation times were observed in oleogels with AW. Oil binding capacity, another important physicochemical property, was found to be on average 56.31% for PA and 63.78% for AA, and 81.29% and 83.73% for AKA and PKA oleogels, respectively (Table 1). However, higher oil binding capacities were determined in the other oleogels, and no statistically significant difference was observed between these samples (*p* > 0.05). Evaluating these results, it can be concluded that the use of AW for olive pomace oil and argan oil oleogelation generally results in lower oil binding capacity, while CW and KW provide more effective oil binding. Similarly, in a study by Blake et al. [8], the oil binding capacity of sunflower wax was found to be lower than that of candelilla and carnauba waxes. Shi et al. [24] determined the oil binding capacity to be 99.70% in oleogels prepared from camellia oil using carnauba wax, and 100% in oleogels prepared using candelilla wax. In another study, it was reported that the oil binding capacity of oleogels prepared using virgin olive oil and different proportions of carnauba wax varied depending on the wax ratios used, and the oil binding capacity of the oleogel using 10% carnauba wax was determined as 93.41% on average [21].

Palmitic acid and oleic acid contents of argan and olive pomace oils were found to be 12.95 ± 0.40% and 43.27 ± 0.17%, 15.17 ± 0.08% and 68.62 ± 0.01%, respectively (*p* < 0.05). On the other hand, linoleic acid, one of the polyunsaturated fatty acids, was found to be at a higher level in argan oil compared to olive pomace oil (Table S1). Other studies have also determined high levels of oleic (46–48%) and linoleic (31–35%) acids contents in argan oil [14,25]. It was also reported that olive pomace oil has significant potential due to its high oleic acid content [26]. It was found that the fatty acid contents of the oleogelators used in the study showed significant differences (*p* < 0.05) (Table S2). It was observed that palmitic acid (C16:0) and oleic acid (C18:1n9c) were at high levels for all waxes. Linoleic acid (C18:2n6c), one of the polyunsaturated fatty acids, was found to have the highest content in the KW group, with an average of 31.00 ± 0.06% compared to other waxes, and the content was found to be the lowest in the AW group, with an average of 0.09 ± 0.05%. It was observed that the fatty acid profiles of the oleogelators formed with the binary combination within the scope of the study were significantly affected by the waxes used. (*p* < 0.05) (Table S2). Chalapud et al. [27] reported that the wax obtained from sunflower oil has higher amounts of palmitic, oleic and linoleic acids. Another study reported that the palmitic and oleic acid contents of sunflower wax are high [28]. Gao et al. [29] reported that the oleic and linoleic acid contents of carnauba wax are high in the fatty acid profile.

It was observed that palmitic, oleic and linoleic acid contents were generally higher in the prepared oleogels (Table S3). While palmitic acid content did not show a statistically significant difference among the oleogels (*p* > 0.05), stearic acid (C18:0) was found to be significantly different (*p* < 0.05). The highest average oleic acid content was determined to be 70.15 ± 0.41% in PKA oleogel, and the lowest average was found to be 35.54 ± 2.82% in AKA oleogel. On the other hand, linoleic acid content was determined to be at higher levels in oleogels prepared with argan oil compared to oleogels prepared with olive pomace oil, and the lowest linoleic acid content was found in PKA oleogel (Table S3). Lim et al. [23] reported that the linoleic acid content was high in oleogels prepared using soybean oil and different proportions of carnauba wax, followed by oleic acid content. In the same study, palmitic acid content was also found to be high. In another study, in which the fatty acid profile was determined in oleogels prepared with palm oil and carnauba wax, palmitic acid and oleic acid content were reported to be high [22]. In a study conducted by Hassim et al. [30], palmitic, oleic and linoleic acids were found to be higher in oleogels prepared with different proportions of carnauba and sunflower waxes, both for the waxes used and for all proportions used.

**Figure 2:**
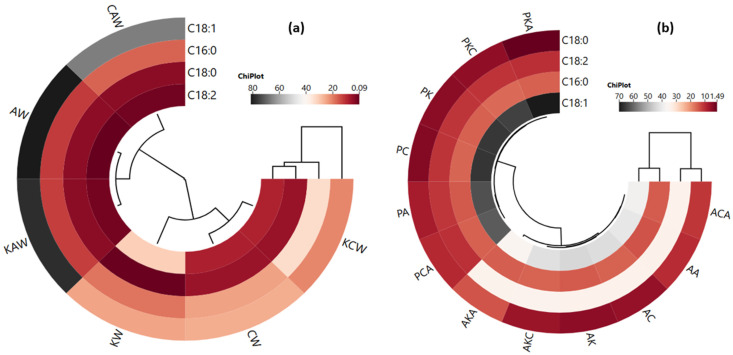
The cluster analysis of a heat map showing the relationship between waxes and major fatty acids (**a**) and between oleogels and major fatty acids (**b**) (AK: argan oil + KW oleogel, PK: olive pomace oil + KW oleogel, AC: argan oil + CW oleogel, PC: olive pomace oil + CW oleogel, AA: argan oil + AW oleogel, PA: olive pomace oil + AW oleogel, AKC: argan oil + KCW oleogel, PKC: olive pomace oil + KCW oleogel, AKA: argan oil + KAW oleogel, PKA: olive pomace oil + KAW oleogel, ACA: argan oil + CAW oleogel, PCA: olive pomace oil + CAW oleogel).

The cluster analysis of the heat map showing the relationship between waxes and major fatty acids (a) and between oleogels and major fatty acids (b) is shown in Figure 2. As can be seen from Figure 2a, two clusters are formed: CAW, AW and KAW in one cluster, and KW, CW and KCW in another cluster. According to this result, sunflower wax differs from the other two waxes in terms of major fatty acids. In addition, oleic acid content is in a separate cluster, showing a different feature than the other major oils (Figure 2a). On the other hand, oleogels formed with argan and olive pomace oils are also separated from each other by being in two different clusters (Figure 2b). In other words, oleogels containing argan oil and olive pomace oil exhibit a different major fatty acid profile from each other; in particular, there are higher values of oleic acid in the groups where olive pomace oil is used compared to those where argan oil is used. Major fatty acids are divided into two clusters. C18:0 and C18:2 are in one cluster, C16:0 and C18:1 are in another cluster, and they exhibit closer correlations within each other (Figure 2b).

### 2.2. Thermal Properties

**Figure 3:**
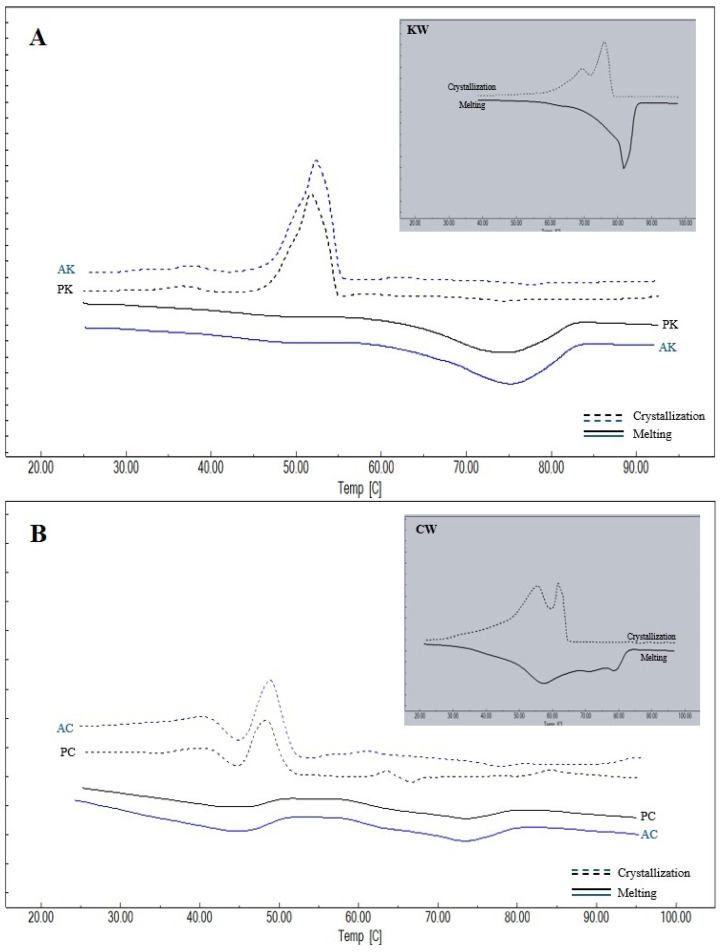

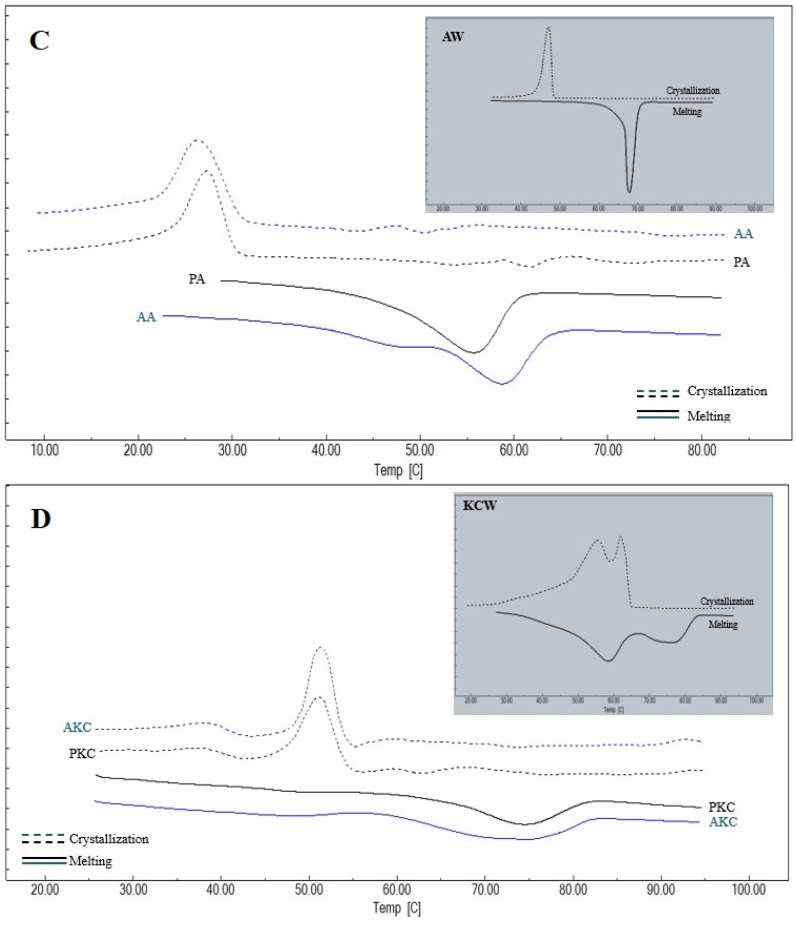

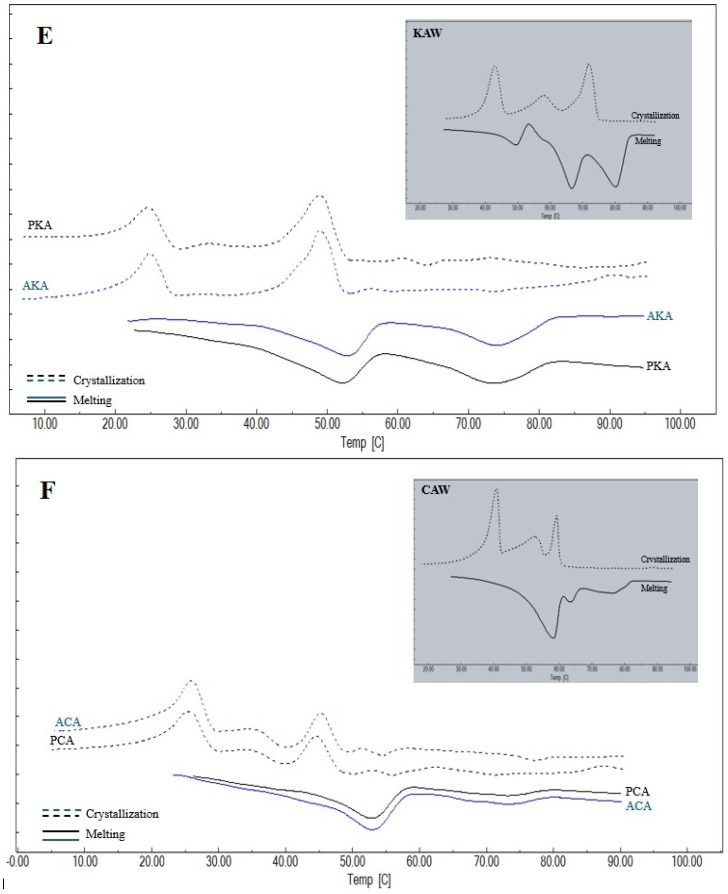
Thermograms of waxes and oleogels (**A**) KW: carnauba wax, AK: argan oil + KW, PK: olive pomace oil + KW; (**B**) CW: candelilla wax, AC: argan oil + CW, PC: olive pomace oil + CW; (**C**) AW: sunflower wax, PA: olive pomace oil + AW, AA: argan oil + AW; (**D**) KCW: %50 carnauba + %50 candelilla waxes, AKC: argan oil + KCW, PKC: olive pomace oil + KCW; (**E**) KAW: %50 carnauba + %50 sunflower waxes, PKA: olive pomace oil + KAW, AKA: argan oil + KAW; (**F**) CAW: %50 candelilla + %50 sunflower waxes, ACA: argan oil + CAW, PCA: olive pomace oil + CAW.

The thermal behavior of the waxes and oleogels provides critical insights into their crystal network structure and component compatibility. Thermograms of the melting and crystallization properties of the oleogels prepared and the oleogelators used in the study are given in Figure 3, and the melting and crystallization peak temperatures and enthalpy values obtained from the thermograms are given in Table 2 and Table 3. For KW, CW and AW waxes, a single peak temperature was observed both in melting and crystallization. This suggests a relatively high degree of chemical homogeneity, or the ability of their minor components to co-crystallize into a single solid phase. However, two different peak temperatures were detected for KCW, and three different peak temperatures were detected for KAW and CAW. This phenomenon indicates a structural heterogeneity that implies that the resulting oleogel network is a composite of different crystal fractions, which may influence the final texture. It was observed that there were differences at the level of *p* < 0.01 between the Peak-1 and Peak-2 temperatures determined during melting and the Peak-1 temperatures and crystallization enthalpies determined during crystallization of oleogelators, and differences at the level of *p* < 0.05 between the melting enthalpies and Peak-3 temperatures and the Peak-2 and Peak-3 temperatures determined during crystallization. When melting is considered, it can be seen that the highest average Peak-1 temperature among the oleogelators is determined in KW and the lowest temperature is determined in KAW. The higher melting temperature and enthalpy values of KW indicate a crystal network with high thermal stability and dense molecular packing, likely driven by its high content of long-chain esters. Peak-2 temperature for melting is observed only in oleogelator mixtures, and the highest value for this temperature is determined for KCW. Peak-3 temperature is observed only in KAW and CAW oleogelators, and a higher value is obtained for KAW. In terms of the melting enthalpies, the highest value for KAW was found to be 228.54 ± 3.32 J/g. Similarly, the highest crystallization Peak-1 temperature and enthalpy value were determined in KW, followed by the CW oleogelator. Similarly to the present study, Blake et al. [31] also determined a higher melting peak temperature for carnauba wax, followed by sunflower and candelilla waxes, respectively. Öğütcü and Yılmaz [21] reported the crystallization peak temperature of carnauba wax as 75.04 °C and the melting peak temperature as 76.30 °C. In another study, the melting peak temperature and crystallization temperature of sunflower wax were determined as 65.31 °C and 61.78 °C, respectively [32].

A single peak temperature was determined during both melting and crystallization in AK, PK, AA and PA oleogels, while two different peak temperatures were determined in AC, PC, AKC and PKC, and AKA and PKA oleogels. On the other hand, two different peaks were obtained during melting in ACA and PCA oleogels, while three different peak temperatures were determined during crystallization. Notably, the presence of multiple peaks in complex oleogels confirms that the phase separation observed in binary waxes persists within the oil medium. Within the scope of the study, the lowest melting Peak-1 temperatures and enthalpy values were determined in AC and PC oleogels, while the highest values were determined in AK and PK oleogels. In addition, the highest crystallization peak temperatures and enthalpies were found in AK and PK, while lower crystallization temperatures were determined in the groups using AW.

In the study, higher temperatures and enthalpies were determined in oleogels using KW, which has higher melting and crystallization temperatures and enthalpy values than other oleogelators, indicating that the wax used in the preparation of the oleogel greatly affects the thermal properties of the oleogel. High enthalpy values in KW-based oleogels signify a higher amount of crystallized mass and stronger intermolecular forces within the gel network, which directly correlate with the formation of a firmer and more heat-resistant oleogel. There are also studies in which melting and crystallization properties were determined in oleogels formed using carnauba, candelilla or sunflower wax [12,24,32]. Blake et al. [31] determined similar melting and crystallization values in oleogels prepared from canola oil using carnauba, candelilla and sunflower waxes to those in this study. In another study in which thermal properties were determined in oleogels formed from olive oil using carnauba wax, different melting and crystallization temperatures were reported [21], indicating that the thermal properties of oleogels are affected by the thermal properties of both the oil and the oleogelators used.

The thermal behavior differences can be mechanistically explained by the variation in chemical composition and supercooling requirements of the waxes. KW, composed primarily of long-chain wax esters, exhibits a high melting point, creating a high degree of supersaturation upon cooling. This high supersaturation acts as a strong thermodynamic driving force for rapid nucleation, resulting in the formation of a dense crystal network. In contrast, the multi-peak behavior observed in mixed waxes (e.g., KAW, CAW) suggests a fractional crystallization mechanism where incompatible chemical components crystallize separately, potentially creating lattice defects that weaken the overall structure.

### 2.3. Textural Properties

The values of the textural properties of the oleogels prepared within the scope of the research are given in Table 4. It was determined that the hardness, adhesiveness and gumminess values of the oleogels differed at the level of *p* < 0.01, while the cohesiveness and springiness values differed at the level of *p* < 0.05. However, no statistically significant difference was found between the resilience values of the samples (*p* > 0.05). The variations in textural parameters are fundamentally linked to the chemical nature of the oleogelators and the resulting crystal network morphology. While the highest hardness values were determined in the oleogels using the KW oleogelator, the lowest hardness values were determined in the samples using the AW oleogelator. Similar situations are also observed for the adhesiveness and gumminess values. On the other hand, higher values for cohesiveness were obtained in the oleogels using the AW oleogelator. When the springiness value was taken into consideration, higher values were determined in the pomace oil oleogels than in the argan oil oleogels, except for the groups where AW was used alone as an oleogelator. The higher hardness of oleogels containing KW can be attributed to the high content of high-melting-point wax esters in carnauba wax. These esters tend to crystallize into fine, needle-like structures with a high surface-to-volume ratio, facilitating the formation of a dense and highly entangled 3D network that entraps the liquid oil effectively. Conversely, the lower hardness in AW oleogels suggests a different crystal growth mechanism. AW tends to form larger, platelet-like crystals. While effective at oil binding at low concentrations, these platelets can stack parallel to each other, creating slip planes that reduce the macroscopic firmness of the gel. The hardness values obtained provide a direct indicator of suitability for specific food applications. For instance, the hardness of KW oleogels aligns with texture profiles in bakery and confectionery products. In contrast, the softer texture of AW and binary oleogels is comparable to commercial tub margarines and hazelnut spreads, suggesting their utility in applications where spreadability at room temperature is the primary quality attribute. The higher cohesiveness of oleogels containing AW suggests that while the AW crystal network is softer, the internal bonds between the crystal aggregates are sufficiently flexible to maintain structural integrity without fracturing immediately. Öğütcü and Yılmaz [21] reported that the use of carnauba wax in oleogels prepared from virgin olive oil caused higher hardness and adhesiveness values compared to other waxes. In another study in which the hardness, adhesiveness, cohesiveness and gumminess parameters of oleogels obtained from canola oil using candelilla and carnauba waxes were determined, the hardness of the oleogel using carnauba wax was determined as 10.43 ± 0.13 N, and that of the oleogel using candelilla wax was determined as 25.12 ± 2.05 N. It was also reported that adhesiveness and cohesiveness parameters were higher in oleogels using carnauba wax [23]. Shi et al. [24] determined that the use of candelilla wax caused a tighter and more adhesive structure compared to the use of carnauba wax in oleogels prepared with both camellia oil and medium-chain triglycerides.

**Figure 4:**
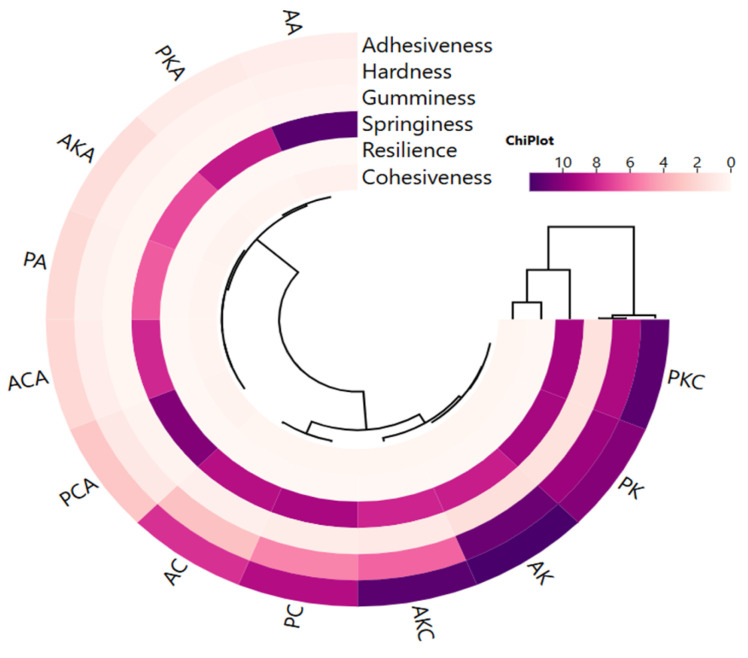
The cluster analysis of a heat map showing the relationship between oleogels and textural properties (AK: argan oil + KW oleogel, PK: olive pomace oil + KW oleogel, AC: argan oil + CW oleogel, PC: olive pomace oil + CW oleogel, AA: argan oil + AW oleogel, PA: olive pomace oil + AW oleogel, AKC: argan oil + KCW oleogel, PKC: olive pomace oil + KCW oleogel, AKA: argan oil + KAW oleogel, PKA: olive pomace oil + KAW oleogel, ACA: argan oil + CAW oleogel, PCA: olive pomace oil + CAW oleogel).

Adhesiveness, hardness and gumminess showed a close correlation with each other, while springiness, resilience and cohesiveness were in a different cluster and showed a close correlation with each other (Figure 4). Two main clusters were distinguished in oleogels. It is seen that oleogels using sunflower wax are different in terms of texture profile from oleogels formed with carnauba and candelilla waxes. However, the use of argan or olive pomace oil does not cause a significant difference in texture properties (Figure 4). According to this result, the textural properties of oleogels are affected by the wax used rather than the oil used in preparation.

## 3. Conclusions

This study confirmed that carnauba (KW), candelilla (CW) and sunflower (AW) waxes are effective structurants for argan and olive pomace oils, with the type of wax playing the dominant role in determining the final oleogel properties. KW has emerged as the superior oleogelator, producing gels with high thermal stability, oil binding capacity and hardness due to its ability to form a dense crystal network. In contrast, samples containing AW exhibited structural heterogeneity and significantly lower hardness and reduced oxidative stability, driven by the poor initial quality of the wax. From an application perspective, this study confirms that olive pomace oil can be successfully valorized into a cost-effective solid fat replacer, potentially increasing the economic value of this byproduct. Simultaneously, argan oil oleogels offer a novel route to deliver essential fatty acids and bioactive compounds in a solid format, suitable for functional food applications. Consequently, KW-based oleogels of both oils present promising, zero-trans-fat alternatives for the food industry, with specific potential in bakery and confectionery products where high thermal stability and hardness are required. However, further research is necessary to evaluate the storage stability, sensory profiles, and performance of these oleogels in model food systems.

## 4. Materials and Methods

### 4.1. Materials

The edible argan oil used in the oleogelation was supplied by Jibal Azyar in Agadir, Morocco. Olive pomace oil was obtained from a national commercial company (Verde, Türkiye). The carnauba, candelilla and sunflower waxes were purchased from a national commercial company (Sabunaria-MSA A.Ş., Ankara, Türkiye).

### 4.2. Preparation of Oleogels

**Figure 5:**
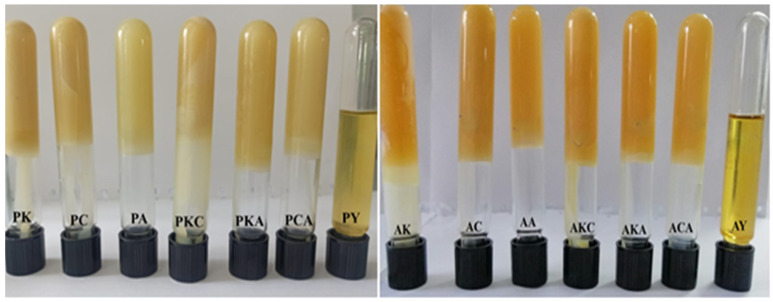
Physical appearance of oils and oleogel (AY: argan oil, PY: olive pomace oil, AK: argan oil + KW oleogel, PK: olive pomace oil + KW oleogel, AC: argan oil + CW oleogel, PC: olive pomace oil + CW oleogel, AA: argan oil + AW oleogel, PA: olive pomace oil + AW oleogel, AKC: argan oil + KCW oleogel, PKC: olive pomace oil + KCW oleogel, AKA: argan oil + KAW oleogel, PKA: olive pomace oil + KAW oleogel, ACA: argan oil + CAW oleogel, PCA: olive pomace oil + CAW oleogel).

The oleogels were prepared using the method described by Choi et al. [33]. Oleogelators were melted in a water bath at 90 °C in single or double combinations (KW: carnauba wax, CW: candelilla wax, AW: sunflower wax, KCW: 50% carnauba + 50% candelilla wax, KAW: 50% carnauba + 50% sunflower wax, CAW: 50% candelilla + 50% sunflower wax) and then mixed with olive pomace oil or argan oil at a ratio of 10% (*w*/*w*) at the same temperature until a homogeneous distribution was obtained. After this treatment, mixtures were vortexed and kept in a dark environment for 24 h at 25°C. The physical appearance of oils and oleogels is shown in Figure 5. Physicochemical, thermal and textural analyses were performed on samples taken from oleogels. Additionally, some physicochemical and thermal analyses were carried out on olive pomace and argan oils and oleogelators.

### 4.3. Physicochemical Analyses

The color intensities of the samples were determined in three dimensions (L*, a* and b*) using a colorimeter device (CR-400, Minolta Co, Osaka, Japan) according to the criteria given by CIE (Commission Internationale de l’Eclairage). The method proposed by Dassanayake et al. [34] was used as the basis for determining the crystal formation time. To determine the oil binding capacity of the oleogels we used the method provided by Fayaz et al. [35]. The methyl esters of fatty acids were prepared according to the method provided by Metcalfe and Schmitz [36], and the fatty acid compositions were determined using GC/FID (Agilent Technologies, 7820A, Santa Clara, CA, USA). The peroxide analysis was performed according to the method provided by AOCS [37], and the values were given as meq O_2_/kg oil.

### 4.4. Thermal Analyses

A differential scanning calorimetry (DSC-60; Shimadzu Corporation, Kyoto, Japan) was used to determine thermal properties of oleogels and oleogelators. Approximately 3 mg of sample was weighed into an aluminum DSC dish and, using an empty dish as a reference, was first heated to 100 °C at a heating rate of 5 °C/min and then cooled to 0 °C at a cooling rate of 5 °C/min. Nitrogen gas was used as the ambient atmosphere for the measurements and the flow rate was set to at 50 mL/min. The temperature and enthalpy values were determined in the peaks observed from the thermograms obtained as a result of the analyses.

### 4.5. Texture Analysis

Texture profile analysis (TPA) of the oleogels was performed at room temperature with two compression cycles using a texture analyzer (CT3, Brookfield Engineering Laboratories, Middleboro, MA, USA) and a cylindrical probe with a diameter of 12.7 mm (TA 10, Brookfield Engineering Laboratories, USA). In the analysis, the test speed was set to 1 mm/s, the compression distance to 10 mm and the recovery time to as 5 s. Hardness, adhesiveness, resilience, cohesiveness, springiness and gumminess values were calculated from the force versus time curves obtained.

### 4.6. Statistical Analysis

The oil and the oleogelator were considered as factors, and the experiments were performed according to a 2 × 6 factorial design with a completely randomized plan. The data obtained from the study were subjected to variance analysis, and the means of the main sources of variation found to be significant were compared using the Duncan multiple comparison test (IBM SPSS Statistics 20). Cluster analysis of a heat map was also performed using a chi-plot program to determine the relationship between waxes, oleogels and color parameters, between waxes, oleogels and important fatty acids and between texture properties and oleogels. In the algorithm, complete is performed as the method and correlation is taken as the distance (https://www.chiplot.online).

## Figures and Tables

**Table 1:** Color properties, peroxide values, crystallization formation and oil binding capacity of oleogels and waxes (mean ±: standard error).

Waxes	L*	a*	b*	Peroxide Value (meq O_2_)	Crystallization Formation (s)	
KW	69.13 ± 0.59 ^c^	3.57 ± 0.25 ^d^	43.87 ± 0.07 ^e^	12.26 ± 0.02 ^c^	240 ± 0.00 ^b^	
CW	62.63 ± 1.17 ^b^	3.71 ± 0.23 ^d^	37.17 ± 0.16 ^d^	1.99 ± 0.01 ^a^	330 ± 0.00 ^c^	
AW	75.91 ± 1.18 ^d^	−2.93 ± 0.05 ^a^	1.51 ± 0.13 ^a^	134.93 ± 0.47 ^f^	840 ± 0.00 ^e^	
KCW	55.09 ± 0.44 ^a^	4.08 ± 0.07 ^d^	29.97 ± 0.37 ^c^	7.55 ± 0.01 ^b^	240 ± 0.00 ^b^	
KAW	56.27 ± 0.79 ^a^	2.45 ± 0.44 ^c^	29.64 ± 0.17 ^c^	79.80 ± 0.07 ^e^	410 ± 0.00 ^d^	
CAW	61.17 ± 1.11 ^b^	−0.39 ± 0.13 ^b^	26.20 ± 0.14 ^b^	73.49 ± 0.09 ^d^	210 ± 0.00 ^a^	
Significance	**	**	**	**	**	
**Oleogels**	L*	a*	b*	**Peroxide value (meq O_2_)**	**Crystallization** **Formation (s)**	**Oil Binding** **Capacity (%)**
AK	47.08 ± 0.21 ^c^	−2.15 ± 0.06 ^e^	16.93 ± 0.49 ^ab^	1.78 ± 0.01 ^b^	540 ± 0.00 ^a^	97.86 ± 0.02 ^d^
PK	51.17 ± 0.01 ^d^	−4.97 ± 0.00 ^d^	20.59 ± 0.09 ^bc^	7.05 ± 0.28 ^ef^	570 ± 0.00 ^b^	97.51 ± 0.70 ^d^
AC	62.48 ± 0.34 ^f^	−1.31 ± 0.00 ^f^	22.35 ± 0.35 ^cd^	0.20 ± 0.00 ^a^	720 ± 0.00 ^e^	100 ± 0.00 ^d^
PC	65.61 ± 0.97 ^g^	−6.04 ± 0.01 ^b^	19.07 ± 4.16 ^bc^	4.83 ± 0.48 ^c^	650 ± 0.00 ^c^	100 ± 0.00 ^d^
AA	37.31 ± 0.29 ^a^	−0.14 ± 0.12 ^h^	14.79 ± 0.09 ^a^	11.81 ± 0.02 ^h^	2280 ± 0.00 ^l^	63.78 ± 2.65 ^b^
PA	41.99 ± 0.91 ^b^	−5.79 ± 0.22 ^bc^	14.69 ± 0.22 ^a^	12.41 ± 0.21 ^hi^	2250 ± 0.00 ^k^	56.31 ± 1.33 ^a^
AKC	55.41 ± 1.09 ^e^	−2.17 ± 0.10 ^e^	19.41 ± 0.25 ^bc^	0.89 ± 0.09 ^ab^	780 ± 0.00 ^f^	98.00 ± 0.63 ^d^
PKC	55.85 ± 1.15 ^e^	−5.71 ± 0.03 ^c^	19.50 ± 0.12 ^bc^	6.03 ± 0.09 ^d^	675 ± 0.00 ^d^	100 ± 0.00 ^d^
AKA	63.29 ± 0.03 ^f^	−0.52 ± 0.04 ^g^	29.79 ± 0.95 ^e^	6.29 ± 0.19 ^de^	1410 ± 0.00 ^j^	81.29 ± 0.19 ^c^
PKA	67.43 ± 0.13 ^g^	−5.98 ± 0.02 ^bc^	31.83 ± 0.51 ^e^	10.52 ± 0.21 ^g^	955 ± 0.00 ^h^	83.73 ± 0.51 ^c^
ACA	66.48 ± 0.19 ^g^	−1.02 ± 0.15 ^f^	25.65 ± 0.83 ^d^	7.49 ± 0.01 ^f^	1110 ± 0.00 ^i^	98.26 ± 0.64 ^d^
PCA	55.98 ± 0.19 ^e^	−6.55 ± 0.10 ^a^	20.93 ± 0.08 ^bc^	13.10 ± 0.81 ^i^	900 ± 0.00 ^g^	100 ± 0.00 ^d^
Significance	**	**	**	**	**	**

KW: carnauba wax, CW: candelilla wax, AW: sunflower wax, KCW: %50 carnauba + %50 candelilla waxes, KAW: %50 carnauba + %50 sunflower waxes, CAW: %50 candelilla + %50 sunflower waxes, AK: argan oil + KW oleogel, PK: olive pomace oil + KW oleogel, AC: argan oil + CW oleogel, PC: olive pomace oil + CW oleogel, AA: argan oil + AW oleogel, PA: olive pomace oil + AW oleogel, AKC: argan oil + KCW oleogel, PKC: olive pomace oil + KCW oleogel, AKA: argan oil + KAW oleogel, PKA: olive pomace oil + KAW oleogel, ACA: argan oil + CAW oleogel, PCA: olive pomace oil + CAW oleogel; ^a–l^: Means marked with different letters in the same column are statistically different from each other (*p* < 0.05); ** *p* < 0.01.

**Table 2:** Thermal properties of waxes (mean ±: standard error).

	Melting				Crystallization			
	Peak-1	Peak-2	Peak-3	Heat-1	Peak-1	Peak-2	Peak-3	Heat-1
KW	81.79 ± 0.06 ^e^	-	-	228.54 ± 3.32 ^c^	75.98 ± 0.01 ^f^	-	-	206.07 ± 4.33 ^c^
CW	57.37 ± 0.08 ^b^	-	-	214.61 ± 7.65 ^bc^	61.70 ± 0.05 ^e^	-	-	202.97 ± 6.85 ^c^
AW	67.48 ± 0.14 ^d^	-	-	183.29 ± 13.38 ^a^	46.95 ± 0.03 ^c^	-	-	117.10 ± 6.04 ^a^
KCW	58.85 ± 0.31 ^c^	76.57 ± 0.26 ^c^	-	194.55 ± 4.76 ^ab^	55.69 ± 0.07 ^d^	61.96 ± 0.02 ^b^	-	192.41 ± 4.87 ^bc^
KAW	49.53 ± 0.08 ^a^	66.35 ± 0.31 ^b^	79.81 ± 0.36 ^b^	199.82 ± 1.18 ^ab^	42.83 ± 0.16 ^b^	58.54 ± 0.55 ^b^	71.79 ± 0.15 ^b^	180.73 ± 4.26 ^b^
CAW	57.37 ± 0.23 ^b^	63.17 ± 0.01 ^a^	76.35 ± 0.65 ^a^	189.25 ± 4.30 ^ab^	38.87 ± 1.45 ^a^	53.71 ± 1.53 ^a^	60.21 ± 1.56 ^a^	176.77 ± 1.71 ^b^
Significance	**	**	*	*	**	*	*	**

KW: carnauba wax, CW: candelilla wax, AW: sunflower wax, KCW: %50 carnauba + %50 candelilla waxes, KAW: %50 carnauba + %50 sunflower waxes, CAW: %50 candelilla + %50 sunflower waxes, ^a–f^: Means marked with different letters in the same column are statistically different from each other (*p* < 0.05); ** *p* < 0.01, * *p* < 0.05.

**Table 3:** Thermal properties of oleogels (mean ±: standard error).

	Melting				Crystallization					
	Peak-1	Heat-1	Peak-2	Heat-2	Peak-1	Heat-1	Peak-2	Heat-2	Peak-3	Heat-3
AK	75.01 ± 0.29 ^f^	20.15 ± 2.81 ^f^	-	-	52.77 ± 0.38 ^g^	19.09 ± 2.55 ^d^	-	-	-	-
PK	74.97 ± 0.29 ^f^	17.85 ± 1.93 ^ef^	-	-	51.75 ± 0.01 ^f^	18.42 ± 1.72 ^d^	-	-	-	-
AC	45.06 ± 0.21 ^a^	5.70 ± 0.19 ^ab^	73.40 ± 0.09 ^a^	4.91 ± 0.45 ^c^	40.63 ± 0.45 ^e^	3.29 ± 0.03 ^ab^	48.75 ± 0.14 ^bc^	5.46 ± 0.33 ^c^	-	-
PC	45.18 ± 0.19 ^a^	2.67 ± 0.05 ^a^	73.51 ± 0.11 ^a^	2.83 ± 0.21 ^b^	39.75 ± 0.37 ^e^	1.81 ± 0.43 ^a^	48.02 ± 0.30 ^b^	3.40 ± 0.08 ^b^	-	-
AA	58.52 ± 0.27 ^e^	16.03 ± 2.09 ^def^	-	-	25.96 ± 0.37 ^b^	13.21 ± 2.03 ^c^	-	-	-	-
PA	55.65 ± 0.05 ^d^	14.41 ± 0.39 ^de^	-	-	27.35 ± 0.13 ^c^	12.96 ± 0.17 ^c^	-	-	-	-
AKC	48.89 ± 0.09 ^b^	12.68 ± 0.11 ^d^	74.68 ± 0.03 ^a^	-	37.96 ± 0.07 ^d^	12.83 ± 0.21 ^c^	51.18 ± 0.15 ^d^	-	-	-
PKC	49.58 ± 0.86 ^b^	12.18 ± 1.77 ^cd^	74.66 ± 0.07 ^a^	-	37.13 ± 0.17 ^d^	12.60 ± 0.81 ^c^	50.79 ± 0.15 ^cd^	-	-	-
AKA	52.84 ± 0.03 ^c^	6.45 ± 0.39 ^ab^	72.57 ± 1.47 ^a^	6.34 ± 0.40 ^d^	25.05 ± 0.19 ^ab^	5.02 ± 0.74 ^ab^	49.14 ± 0.16 ^bcd^	7.19 ± 0.79 ^d^	-	-
PKA	52.39 ± 0.05 ^c^	8.17 ± 0.61 ^bc^	71.90 ± 1.58 ^a^	7.18 ± 0.06 ^d^	24.69 ± 0.05 ^a^	5.43 ± 0.35 ^ab^	48.74 ± 0.19 ^bc^	9.16 ± 0.31 ^e^	-	-
ACA	53.16 ± 0.20 ^c^	8.11 ± 0.53 ^bc^	73.44 ± 0.23 ^a^	1.70 ± 0.16 ^a^	25.28 ± 0.60 ^ab^	6.66 ± 0.92 ^b^	33.79 ± 0.76 ^a^	1.05 ± 0.17 ^a^	44.67 ± 0.59 ^a^	3.06 ± 0.12 ^a^
PCA	52.71 ± 0.31 ^c^	6.59 ± 0.15 ^ab^	73.13 ± 0.11 ^a^	1.84 ± 0.07 ^a^	25.32 ± 0.24 ^ab^	6.33 ± 0.23 ^b^	33.28 ± 1.54 ^a^	1.18 ± 0.05 ^a^	44.43 ± 0.01 ^a^	3.02 ± 0.17 ^a^
Significance	**	**	ns	**	**	**	**	**	ns	ns

AK: argan oil + KW oleogel, PK: olive pomace oil + KW oleogel, AC: argan oil + CW oleogel, PC: olive pomace oil + CW oleogel, AA: argan oil + AW oleogel, PA: olive pomace oil + AW oleogel, AKC: argan oil + KCW oleogel, PKC: olive pomace oil + KCW oleogel, AKA: argan oil + KAW oleogel, PKA: olive pomace oil + KAW oleogel, ACA: argan oil + CAW oleogel, PCA: olive pomace oil + CAW oleogel; ^a–g^: Means marked with different letters in the same column are statistically different from each other (*p* < 0.05); ** *p* < 0.01, ns: not significance.

**Table 4:** Texture properties of oleogels (mean ±: standard error).

	Hardness (N)	Adhesiveness (mJ)	Resilience	Cohesiveness	Springiness (mm)	Gumminess (N)
AK	10.93 ± 0.19 ^h^	11.98 ± 2.86 ^d^	0.010 ± 0.00 ^a^	0.143 ± 0.03 ^a^	8.19 ± 0.34 ^ab^	1.55 ± 0.31 ^e^
PK	9.49 ± 0.16 ^g^	10.17 ± 1.12 ^bcd^	0.008 ± 0.01 ^a^	0.143 ± 0.01 ^a^	9.16 ± 0.94 ^abc^	1.35 ± 0.10 ^e^
AC	3.09 ± 0.05 ^c^	7.60 ± 0.47 ^b^	0.008 ± 0.01 ^a^	0.163 ± 0.01 ^a^	8.71 ± 0.37 ^abc^	0.51 ± 0.03 ^bc^
PC	5.24 ± 0.09 ^d^	8.78 ± 0.55 ^bc^	0.007 ± 0.01 ^a^	0.138 ± 0.01 ^a^	9.09 ± 0.35 ^abc^	0.73 ± 0.03 ^cd^
AA	0.36 ± 0.03 ^a^	0.63 ± 0.30 ^a^	0.010 ± 0.01 ^a^	0.318 ± 0.10 ^b^	11.55 ± 2.37 ^c^	0.17 ± 0.05 ^a^
PA	0.39 ± 0.02 ^a^	1.83 ± 0.53 ^a^	0.010 ± 0.00 ^a^	0.230 ± 0.06 ^ab^	6.36 ± 1.05 ^a^	0.11 ± 0.02 ^a^
AKC	6.15 ± 0.25 ^e^	11.42 ± 0.52 ^cd^	0.010 ± 0.00 ^a^	0.146 ± 0.01 ^a^	8.01 ± 0.43 ^ab^	0.90 ± 0.07 ^d^
PKC	8.99 ± 0.15 ^f^	11.40 ± 0.62 ^cd^	0.007 ± 0.01 ^a^	0.152 ± 0.01 ^a^	9.22 ± 0.61 ^abc^	1.37 ± 0.11 ^e^
AKA	0.38 ± 0.02 ^a^	1.57 ± 0.15 ^a^	0.012 ± 0.01 ^a^	0.270 ± 0.04 ^ab^	6.83 ± 0.40 ^a^	0.11 ± 0.02 ^a^
PKA	0.30 ± 0.02 ^a^	0.82 ± 0.17 ^a^	0.010 ± 0.01 ^a^	0.183 ± 0.05 ^a^	8.34 ± 0.61 ^ab^	0.06 ± 0.02 ^a^
ACA	0.63 ± 0.04 ^a^	1.92 ± 0.12 ^a^	0.008 ± 0.01 ^a^	0.177 ± 0.03 ^a^	7.88 ± 0.31 ^ab^	0.11 ± 0.02 ^a^
PCA	0.96 ± 0.03 ^b^	2.80 ± 0.26 ^a^	0.015 ± 0.01 ^a^	0.275 ± 0.03 ^ab^	10.27 ± 0.55 ^bc^	0.27 ± 0.03 ^ab^
Significance	**	**	ns	*	*	**

AK: argan oil + KW oleogel, PK: olive pomace oil + KW oleogel, AC: argan oil + CW oleogel, PC: olive pomace oil + CW oleogel, AA: argan oil + AW oleogel, PA: olive pomace oil + AW oleogel, AKC: argan oil + KCW oleogel, PKC: olive pomace oil + KCW oleogel, AKA: argan oil + KAW oleogel, PKA: olive pomace oil + KAW oleogel, ACA: argan oil + CAW oleogel, PCA: olive pomace oil + CAW oleogel; ^a–h^: Means marked with different letters in the same column are statistically different from each other (*p* < 0.05); ** *p* < 0.01, * *p* < 0.05, ns: not significance.

## Data Availability

All data and materials are available on request from the corresponding author.

## Supplementary Materials

The following supporting information can be downloaded at https://www.mdpi.com/article/10.3390/gels11120997/s1, Table S1: Fatty acid composition of argan and olive pomace oils (%); Table S2: Fatty acid composition of waxes (%); Table S3: Fatty acid composition of oleogels (%).
