# Supplementary material for: Physicochemical, Thermal and Textural Characterization of Olive Pomace Oil and Argan Oil Oleogels Prepared with Different Oleogelators
Source: Gels. 2025 Dec 11;11(12):997. doi: 10.3390/gels11120997 (PMC12732674; doi:10.3390/gels11120997)
Supplement: Supplementary file 1 [file gels-11-00997-s001.zip › gels-3987651-SI.pdf]

Table Captions

Table S1. Fatty acid composition of argan and olive pomace oils (%)

Table S2. Fatty acid composition of waxes (%)

Table S3. Fatty acid composition of oleogels (%)

Table S1. Fatty acid composition of argan and olive pomace oils (%) (mean ±: standard error)

	**AY**	**PY**	**Significance**
C12:0 Lauric acid	0.01±0.00^{a}	0.01±0.00^{a}	ns
C14:0 Myristic acid	0.21±0.01^{a}	0.09±0.05^{a}	ns
C16:0 Palmitic acid	12.95±0.40^{a}	15.17±0.08^{b}	*
C18:0 Stearic acid	3.59±0.20^{a}	2.75±0.03^{a}	ns
C20:0 Arachidic acid	0.21±0.12^{a}	0.08±0.03^{a}	ns
C21:0 Heneicosanoic acid	0.13±0.06^{a}	0.07±0.02^{a}	ns
C22:0 Behenic acid	0.03±0.01^{a}	0.05±0.01^{a}	ns
C24:0 Lignoceric acid	0.45±0.43^{a}	0.02±0.01^{a}	ns
C14:1 Myristoleic acid	0.06±0.01^{a}	0.03±0.02^{a}	ns
C16:1 (n-7) Palmitoleic acid	0.15±0.01^{a}	1.15±0.01^{b}	**
C18:1 (n-9c) Oleic acid	43.27±0.17^{a}	68.62±0.01^{b}	**
C18:2 (n-6c) Linoleic acid	38.18±0.11^{b}	11.20±0.08^{a}	**
C18:2 (n-6t) Linolelaidic acid	0.36±0.03^{a}	0.36±0.07^{a}	ns
C18:3 α-Linolenic acid	0.27±0.01^{a}	0.35±0.01^{b}	*
C18:3 (n-3c) γ-Linolenic acid	0.07±0.02^{a}	0.02±0.01^{a}	ns
C20:2 Dihomo-linoleic acid	0.04±0.01^{a}	0.05±0.01^{a}	ns
C20:3 (n-3) Dihomo- α -linolenic acid	0.01±0.00^{a}	0.01±0.01^{a}	ns
C20:5 Eicosapentaenoic acid	0.00±0.00^{a}	0.00±0.00^{a}	ns

AY: argan oil, PY: olive pomace oil, $^{a-b:}$ Mean marked with different letters in the same column are statistically different from each other (P<0.05); **p<0.01, * p<0.05, ns: not significance

Table S2. Fatty acid composition of waxes (%) (mean ±: standard error)

	KW	CW	AW	KCW	CAW	KAW	Significance
C12:0 Lauric acid	0.75±0.09^{b}	1.65±0.22^{c}	0.26±0.04^{a}	1.60±0.07^{c}	0.41±0.01^{ab}	0.31±0.04^{a}	**
C14:0 Myristic acid	1.87±0.19^{b}	2.39±0.32^{b}	0.32±0.05^{a}	2.27±0.08^{b}	0.52±0.04^{a}	0.37±0.03^{a}	**
C16:0 Palmitic acid	18.81±0.87^{c}	24.47±0.73^{d}	11.99±0.32^{a}	33.28±0.05^{e}	16.87±0.49^{b}	12.41±0.34^{a}	**
C18:0 Stearic acid	0.32±0.06^{a}	5.84±0.03^{c}	4.17±0.55^{b}	5.64±0.38^{c}	4.13±0.42^{b}	4.08±0.49^{b}	**
C20:0 Arachidic acid	0.23±0.03^{a}	4.98±0.03^{c}	0.23±0.01^{a}	4.96±0.64^{c}	1.45±0.17^{b}	0.05±0.04^{a}	**
C21:0 Heneicosanoic acid	0.61±0.35^{a}	4.52±0.31^{b}	0.74±0.04^{a}	0.86±0.39^{a}	1.54±0.48^{a}	0.92±0.25^{a}	**
C22:0 Behenic acid	0.00±0.00^{a}	1.61±1.61^{a}	0.00±0.00^{a}	4.37±0.86^{b}	0.00±0.00^{a}	0.18±0.18^{a}	*
C24:0 Lignoceric acid	8.71±0.19^{c}	4.03±0.78^{b}	0.00±0.00^{a}	3.49±0.38^{b}	0.63±0.09^{a}	0.55±0.10^{a}	**
C14:1 Myristoleic acid	0.43±0.04^{ab}	1.67±0.23^{b}	0.12±0.02^{a}	1.67±0.05^{b}	3.10±0.87^{c}	0.07±0.01^{a}	*
C16:1 (n-7) Palmitoleic acid	0.14±0.01^{a}	1.99±0.10^{c}	0.04±0.01^{a}	1.34±0.19^{b}	0.00±0.00^{a}	0.00±0.00^{a}	**
C18:1 (n-9c) Oleic acid	24.69±0.05^{ab}	25.86±1.49^{b}	81.44±1.12^{d}	20.91±0.01^{a}	66.28±1.78^{c}	78.35±1.05^{d}	**
C18:2 (n-6c) Linoleic acid	31.00±0.06^{d}	8.09±0.66^{c}	0.09±0.05^{a}	8.62±0.61^{c}	1.31±0.11^{ab}	1.58±0.15^{b}	**
C18:2 (n-6t) Linolelaidic acid	2.20±0.07^{d}	0.99±0.10^{c}	0.04±0.00^{a}	0.38±0.17^{b}	0.02±0.00^{a}	0.02±0.00^{a}	**
C18:3 α-Linolenic acid	0.25±0.13^{ab}	0.45±0.08^{b}	0.05±0.01^{a}	0.13±0.05^{a}	0.08±0.01^{a}	0.05±0.02^{a}	*
C18:3 (n-3c) γ-Linolenic acid	0.24±0.13^{a}	0.09±0.01^{a}	0.19±0.05^{a}	0.30±0.09^{a}	0.18±0.03^{a}	0.14±0.03^{a}	ns
C20:2 Dihomo-linoleic acid	9.40±0.25^{c}	2.80±1.33^{b}	0.20±0.01^{a}	0.54±0.14^{a}	0.41±0.20^{a}	0.90±0.05^{ab}	**
C20:3 (n-3) Dihomo- α -linolenic acid	0.12±0.01^{a}	3.76±0.05^{b}	0.05±0.03^{a}	4.60±1.37^{b}	1.76±0.15^{a}	0.04±0.04^{a}	*
C20:5 Eicosapentaenoic acid	0.22±0.11^{ab}	4.79±0.43^{c}	0.06±0.01^{a}	5.04±0.67^{c}	1.30±0.04^{b}	0.01±0.01^{a}	**

KW: carnauba wax, CW: candellilla wax, AW: sunflower wax, KCW: %50 carnauba + %50 candellilla wax. KAW: %50 carnauba + %50 sunflower wax. CAW: %50 candellilla + %50 sunflower wax, a-f: Mean marked with different letters in the same column are statistically different from each other (P<0.05); **p<0.01,* p<0.05

Table S3. Fatty acid composition of oleogels (%) (mean ±: standard error)

	AK	PK	AC	PC	AA	PA	AKC	PKC	AKA	PKA	ACA	PCA	Sig.
C12:0 Lauric acid	0.01±0.00^{a}	0.01±0.00^{a}	0.05±0.05^{a}	0.01±0.01^{a}	0.02±0.00^{a}	0.02±0.01^{a}	0.01±0.00^{a}	0.01±0.00^{a}	0.01±0.00^{a}	0.01±0.00^{a}	0.01±0.00^{a}	0.01±0.00^{a}	ns
C14:0 Myristic acid	0.17±0.04^{d}	0.04±0.00^{a}	0.21±0.01^{d}	0.05±0.01^{a}	0.21±0.01^{d}	0.11±0.02^{bc}	0.21±0.01^{d}	0.04±0.01^{a}	0.16±0.04^{cd}	0.04±0.01^{a}	0.22±0.01^{d}	0.05±0.00^{ab}	**
C16:0 Palmitic acid	14.76±0.28^{a}	15.35±0.07^{a}	15.47±0.19^{a}	15.72±0.10^{a}	13.97±0.18^{a}	14.64±0.07^{a}	15.54±0.43^{a}	16.22±0.38^{a}	15.06±0.43^{a}	15.35±0.63^{a}	14.92±0.93^{a}	15.39±0.17^{a}	ns
C18:0 Stearic acid	5.03±0.21^{abc}	4.85±1.10^{abc}	5.75±0.29^{abcd}	4.21±0.56^{ab}	9.82±0.03^{cde}	7.72±0.15^{bcd}	6.52±1.11^{abcd}	5.32±0.89^{abc}	14.13±2.54^{e}	1.49±0.06^{a}	11.04±3.37^{de}	9.42±3.02^{bcde}	*
C20:0 Arachidic acid	0.08±0.04^{bc}	0.10±0.01^{bc}	0.11±0.01^{b}	0.07±0.06^{abc}	0.03±0.01^{abc}	0.22±0.02^{c}	0.02±0.01^{abc}	0.02±0.01^{ab}	0.02±0.01^{a}	0.04±0.02^{abc}	0.04±0.02^{abc}	0.30±0.01^{d}	**
C21:0 Heneicosanoic acid	0.08±0.01^{a}	0.08±0.01^{a}	0.08±0.01^{a}	0.07±0.01^{a}	0.04±0.02^{a}	0.04±0.01^{a}	0.03±0.00^{a}	0.15±0.12^{a}	0.22±0.17^{a}	0.08±0.00^{a}	0.05±0.01^{a}	0.11±0.04^{a}	ns
C22:0 Behenic acid	0.10±0.01^{a}	0.12±0.01^{a}	0.05±0.01^{a}	0.12±0.01^{a}	0.02±0.02^{a}	0.06±0.01^{a}	0.03±0.03^{a}	0.15±0.04^{a}	0.06±0.06^{a}	0.09±0.00^{a}	0.04±0.01^{a}	0.14±0.01^{a}	ns
C24:0 Lignoceric acid	0.09±0.01^{a}	0.09±0.01^{a}	0.04±0.01^{a}	0.07±0.02^{a}	0.01±0.01^{a}	0.09±0.08^{a}	0.06±0.01^{a}	0.06±0.01^{a}	0.05±0.01^{a}	0.04±0.01^{a}	0.02±0.01^{a}	0.05±0.01^{a}	ns
C14:1 Myristoleic acid	0.07±0.00^{c}	0.01±0.01^{ab}	0.08±0.02^{c}	0.02±0.01^{ab}	0.07±0.01^{c}	0.03±0.01^{b}	0.07±0.01^{c}	0.01±0.00^{ab}	0.03±0.01^{ab}	0.22±0.01^{a}	0.08±0.01^{c}	0.01±0.01^{ab}	**
C16:1 (n-7) Palmitoleic acid	0.11±0.03^{a}	1.10±0.01^{b}	0.15±0.01^{a}	1.18±0.01^{bc}	0.14±0.01^{a}	1.13±0.02^{b}	0.09±0.03^{a}	1.20±0.03^{bc}	0.13±0.09^{a}	1.57±0.33^{c}	0.12±0.03^{a}	1.35±0.25^{bc}	**
C18:1 (n-9c) Oleic acid	44.27±0.05^{b}	66.32±1.04^{cd}	43.11±0.19^{b}	66.51±0.27^{cd}	40.96±0.45^{b}	63.10±0.51^{c}	42.49±0.78^{b}	65.01±0.61^{cd}	35.54±2.82^{a}	70.15±0.41^{d}	38.73±3.72^{ab}	61.67±3.01^{c}	**
C18:2 (n-6c) Linoleic acid	34.11±0.11^{c}	10.91±0.09^{ab}	34.07±0.18^{c}	10.85±0.01^{ab}	33.86±0.02^{c}	11.00±0.03^{b}	34.53±0.10^{c}	10.76±0.07^{ab}	34.25±0.26^{c}	10.30±34.12^{a}	34.12±0.62^{c}	10.43±0.09^{ab}	**
C18:2 (n-6t) Linolelaidic acid	0.52±0.01^{d}	0.28±0.23^{bcd}	0.33±0.01^{cd}	0.15±0.03^{abc}	0.25±0.01^{abc}	0.14±0.07^{abc}	0.02±0.01^{a}	0.04±0.01^{ab}	0.01±0.01^{a}	0.01±0.01^{a}	0.03±0.02^{ab}	0.07±0.01^{ab}	*
C18:3 α-Linolenic acid	0.39±0.02^{b}	0.66±0.02^{c}	0.32±0.03^{b}	0.84±0.04^{de}	0.32±0.02^{b}	0.93±0.03^{e}	0.29±0.00^{ab}	0.83±0.03^{de}	0.15±0.15^{a}	0.75±0.03^{cd}	0.27±0.02^{ab}	0.83±0.05^{de}	**
C18:3 (n-3c)) γ-Linolenic acid	0.06±0.01^{b}	0.03±0.01^{ab}	0.05±0.01^{ab}	0.03±0.01^{ab}	0.03±0.01^{ab}	0.10±0.01^{c}	0.02±0.01^{a}	0.05±0.02^{ab}	0.03±0.01^{ab}	0.04±0.00^{ab}	0.05±0.01^{ab}	0.05±0.01^{ab}	**
C20:2 Dihomo-linoleic acid	0.03±0.00^{a}	0.02±0.01^{a}	0.04±0.01^{a}	0.05±0.01^{a}	0.10±0.01^{a}	0.09±0.02^{a}	0.02±0.01^{a}	0.09±0.02^{a}	0.10±0.03^{a}	0.03±0.01^{a}	0.05±0.04^{a}	0.06±0.02^{a}	ns
C20:3 (n-3) Dihomo- α -linolenic acid	0.01±0.00^{a}	0.01±0.01^{a}	0.01±0.01^{a}	0.04±0.01^{b}	0.01±0.00^{a}	0.01±0.00^{a}	0.01±0.01^{a}	0.03±0.01^{b}	0.01±0.01^{a}	0.01±0.00^{a}	0.01±0.00^{a}	0.03±0.01^{b}	**
C20:5 Eicosapentaenoic acid	0.12±0.03^{a}	0.03±0.01^{a}	0.09±0.01^{a}	0.03±0.01^{a}	0.15±0.14^{a}	0.56±0.33^{a}	0.01±0.01^{a}	0.01±0.01^{a}	0.02±0.01^{a}	0.01±0.00^{a}	0.17±0.01^{a}	0.02±0.01^{a}	ns

AK: argan oil + KW oleogel, PK: oil pomace oil + KW oleogel, AC: argan oil + CW oleogel, PC: oil pomace oil + CW oleogel, AA: argan oil + AW oleogel, PA: oil pomace oil + AW oleoge, AKC: argan oil + KCW oleogel, PKC: oil pomace oil + KCW oleogel, AKA: argan oil + KAW oleogel, PKA: oil pomace oil + KAW oleogel, ACA: argan oil + CAW oleogel, PCA: oil pomace oil + CAW oleogel; a-h: Mean marked with different letters in the same column are statistically different from each other (P<0.05); **p<0.01, *p<0.05, ns: not significance
